# Severe Rhabdomyolysis in the Setting of Influenza A Infection and Clinically Suspected Anti-signal Recognition Particle-Positive Immune-Mediated Necrotizing Myopathy

**DOI:** 10.7759/cureus.115167

**Published:** 2026-08-25

**Authors:** Yen Nhi N Hoang, Shikshya Baral

**Affiliations:** 1 Medical School, Edward Via College of Osteopathic Medicine, Blacksburg, USA; 2 Internal Medicine, Sentara Northern Virginia Medical Center, Woodbridge, USA

**Keywords:** anti-signal recognition particle, immune mediated necrotizing myopathy (imnm), immune-mediated necrotizing myopathy (imnm), immune myositis, influenza virus type a, non-traumatic rhabdomyolysis

## Abstract

Anti-signal recognition particle (SRP)-positive immune-mediated necrotizing myopathy (IMNM) is a rare autoimmune myopathy characterized by symmetric proximal weakness, markedly elevated creatine kinase levels, and muscle fiber necrosis with minimal inflammation. A previously healthy 23-year-old man presented with influenza-like symptoms and intractable gastrointestinal symptoms. Testing was positive for influenza A, and initial evaluation demonstrated severe rhabdomyolysis, with a creatine kinase level of 21,694 U/L, oliguric acute kidney injury, and major electrolyte abnormalities. The clinical course progressed to renal failure requiring continuous renal replacement therapy followed by hemodialysis, metabolic encephalopathy secondary to uremia, acute pancreatitis, anemia requiring transfusion, left upper-extremity deep venous thrombosis, and persistent symmetric extremity weakness. A myositis-specific antibody panel was positive for anti-SRP antibodies at 20 SI (reference range, <11 SI), and broad infectious and autoimmune testing was otherwise unrevealing. The patient received high-dose intravenous methylprednisolone for three days, with substantial improvement in strength and a decline in creatine kinase from a peak of 121,627 U/L to 227 U/L. Although mild weakness persisted at discharge, urine output recovered, and hemodialysis was discontinued. This presentation emphasizes that IMNM should be considered in young patients presenting with severe nontraumatic rhabdomyolysis and weakness, particularly when the clinical course is disproportionate to an apparent viral trigger. Early recognition may expedite targeted immunotherapy and multidisciplinary follow-up.

## Introduction

Anti-signal recognition particle (SRP) antibody-positive immune-mediated necrotizing myopathy (IMNM) is one of three principal IMNM subtypes, alongside anti-3-hydroxy-3-methylglutaryl-coenzyme A reductase-positive and seronegative disease. Anti-SRP antibodies are useful for diagnosis and may correlate with disease activity, creatine kinase levels, and degree of muscle weakness [1-3]. Commonly recognized triggers for increased disease activity include viral infections, malignancy, statin drugs (anti-HMGCR myopathy), and genetic predisposition [4].

Patients typically develop acute or subacute symmetric proximal weakness, often affecting the lower extremities more prominently than the upper extremities, with creatine kinase levels frequently exceeding 1,000 U/L [5]. Muscle biopsy classically demonstrates myonecrosis and myoregeneration with minimal inflammatory infiltrate and variable fatty replacement [6]. Myalgias, neck weakness, dysphagia, and dyspnea may occur, while extramuscular involvement, including interstitial lung disease and cardiac manifestations, is less common [7]. Renal involvement in inflammatory myopathies remains incompletely characterized, particularly with respect to its incidence and long-term outcomes. When present, the most common manifestation is acute kidney injury resulting from myoglobinuria-induced acute tubular necrosis [8].

A case series of 100 patients found that anti-SRP-positive IMNM typically presents between 40 and 60 years of age. Younger age at presentation has been associated with greater disease severity and a higher risk of refractory disease despite standard treatment [9,10]. Diagnosis of anti-SRP-positive IMNM relies on the clinical presentation, supportive serologic findings, and exclusion of alternative conditions with overlapping histopathologic features [11]. The immunologic evaluation should include myositis-specific and myositis-associated antibodies. Electromyography and nerve conduction studies may also help distinguish myopathic disease from neurogenic causes of weakness [12]. Muscle biopsy and magnetic resonance imaging of the proximal lower extremities can further differentiate anti-SRP-positive IMNM from dermatomyositis, polymyositis, and anti-3-hydroxy-3-methylglutaryl-coenzyme A reductase-associated IMNM, as the former may demonstrate more diffuse muscle involvement and early fatty infiltration [13].

Anti-SRP-positive IMNM is often refractory to conventional treatment, and corticosteroid monotherapy is rarely sufficient to control disease activity. Most patients therefore require combination immunotherapy, typically including high-dose corticosteroids, an oral immunosuppressive agent, and intravenous immunoglobulin [14]. For refractory disease, B-cell-directed therapies such as rituximab may be considered, with emerging reports describing the use of belimumab and ofatumumab [15,16].

Viral infections, including influenza A, can cause systemic complications such as viral myositis, rhabdomyolysis, acute kidney injury, myocarditis, and systemic inflammatory responses. Viral illness may also serve as a potential trigger or unmasking event for autoimmune disease. This overlap can complicate the initial distinction between isolated viral rhabdomyolysis and an underlying immune-mediated myopathy, particularly when severe muscle enzyme elevation and weakness persist despite appropriate supportive and antiviral treatment. Although influenza-associated rhabdomyolysis has been reported in rare cases, it is more commonly described in pediatric or elderly patients and typically presents with mild to moderate elevations in creatine kinase [17]. We describe an unusual case of a previously healthy young adult with influenza A infection, fulminant rhabdomyolysis, dialysis-requiring acute kidney injury, and anti-SRP antibody positivity, raising concern for probable IMNM.

## Case presentation

This case describes a 23-year-old African American man with no significant medical history who presented to the emergency department with three days of fever, body aches, headache, diarrhea, vomiting, and intractable nausea. He was not taking medications and denied autoimmune disease, sick contacts, or recent trauma. He appeared mildly ill, but initial examination was otherwise unremarkable. Exam was negative for focal neurologic, musculoskeletal, or dermatologic findings. Vital signs showed blood pressure of 135/83 mmHg, heart rate of 103 beats/minute, respiratory rate of 20 breaths/minute, temperature of 36.7 °C, and oxygen saturation of 100% on room air. Polymerase chain reaction testing was positive for influenza A and negative for SARS-CoV-2.

Initial laboratory testing showed severe rhabdomyolysis, with creatine kinase of 21,694 U/L, acute kidney injury with blood urea nitrogen of 28 mg/dL and creatinine of 2.5 mg/dL, hypocalcemia, and hyperphosphatemia (Table 1). Urinalysis showed dark brown, slightly turbid urine. The patient was admitted to inpatient medicine for management of rhabdomyolysis, oliguric acute kidney injury, and electrolyte derangements.

**Table 1:** Laboratory results at emergency department presentation U/L: units per liter; mg/dL: milligrams per deciliter; mL/min/1.73 m²: milliliters per minute per 1.73 meters squared; mmol/L: millimoles per liter

Laboratory test	Presenting value	Reference range
Creatine kinase	21,694 U/L	<149 U/L
Aldolase	1,002.3 U/L	1.2-7.6 U/L
Aspartate aminotransferase	1,220 U/L	10-31 U/L
Alanine aminotransferase	164 U/L	10-31 U/L
Alkaline phosphatase	86 U/L	30-120 U/L
Lactate dehydrogenase	12,092 U/L	135-225 U/L
Blood urea nitrogen	28 mg/dL	6-24 mg/dL
Creatinine	2.5 mg/dL	0.66-1.09 mg/dL
Estimated glomerular filtration rate	32.9 mL/min/1.73 m²	>90 mL/min/1.73 m²
Sodium	135 mmol/L	135-145 mmol/L
Potassium	3.5 mmol/L	3.5-5.0 mmol/L
Calcium	6.1 mg/dL	8.6-10.3 mg/dL
Phosphate	11 mg/dL	2.5-4.5 mg/dL

Although initially hemodynamically stable, the patient rapidly deteriorated and was transferred to the intensive care unit for continuous renal replacement therapy (CRRT) and aggressive electrolyte repletion. He developed severe anion-gap metabolic acidosis and acute metabolic encephalopathy attributed to uremia, both of which rapidly improved. The patient experienced progressive renal failure that later required intermittent hemodialysis. During this period, he developed profound fatigue and bilateral proximal weakness of the upper and lower extremities without paresthesias or radicular symptoms. Muscle strength was scaled at a Medical Research Council (MRC) muscle strength grade 3/5 in the upper extremity and 2/5 in the lower extremity, with notable deficits in palmar grasp and ambulation.

To evaluate the etiology of this presentation of nontraumatic rhabdomyolysis, targeted infectious testing, autoimmune, vasculitis, and myositis-specific laboratory evaluations were performed (Table 2). Human immunodeficiency virus and hepatitis B testing were negative. The extended myositis-specific antibody panel was positive for antibodies against SRP with a signal intensity of 20 (reference range, <11 SI).

**Table 2:** Diagnostic laboratory evaluation for nontraumatic rhabdomyolysis SI: signal intensity; mg/dL: milligrams per deciliter; AI: antibody index; IU/mL: international units per milliliter; SRP: signal recognition particle; PL-7: threonyl-transfer RNA synthetase; PL-12: alanyl-transfer RNA synthetase; EJ: glycyl-transfer RNA synthetase; OJ: isoleucyl-transfer RNA synthetase; Mi-2 alpha: chromodomain helicase DNA-binding protein 3; Mi-2 beta: chromodomain helicase DNA-binding protein 4; MDA5: melanoma differentiation-associated gene 5; TIF1-gamma: transcription intermediary factor 1 gamma; NXP2: nuclear matrix protein 2; HMGCR: 3-hydroxy-3-methylglutaryl-coenzyme A reductase; cN1A: cytosolic 5′-nucleotidase 1A; SSA/Ro: Sjögren syndrome-related antigen A; SSB/La: Sjögren syndrome-related antigen B; Jo-1: histidyl-transfer RNA synthetase; RNP: ribonucleoprotein; Scl-70: scleroderma-70; Sm: Smith; CENP-B: centromere protein B; dsDNA: double-stranded DNA; MPO: myeloperoxidase; PR3: proteinase 3; ANCA: anti-neutrophil cytoplasmic antibody; anti-GBM: anti-glomerular basement membrane; HIV: human immunodeficiency virus

Category	Test	Result	Reference range
Myositis-specific	Signal recognition particle (SRP) antibody	20 SI	<11 SI
Myositis-specific	PL-7, PL-12, EJ, OJ, Mi-2 alpha, Mi-2 beta, MDA5, TIF1-gamma, NXP2 antibodies	<11 SI	<11 SI
Myositis-specific	HMGCR antibody	<2 SI	<11 SI
Myositis-specific	cN1A antibody	<5 SI	<11 SI
Complement studies	Complement component 3	143 mg/dL	83-177 mg/dL
Complement studies	Complement component 4	32 mg/dL	10-40 mg/dL
Autoimmune	SSA/Ro, SSB/La, Jo-1, RNP, Scl-70, Sm, CENP-B, anti-chromatin antibodies	<1.0 AI	<1.0 AI
Autoimmune	Anti-dsDNA antibody	2 IU/mL	<=4 IU/mL
Autoimmune-vasculitis	MPO, PR3, ANCA, anti-GBM antibodies	<1.0 AI	<1.0 AI
Infectious	HIV-1/2 antigen/antibody with reflex	Nonreactive	Nonreactive
Infectious	Hepatitis B surface antigen	Nonreactive	Nonreactive

The clinical constellation of sudden symmetric extremity weakness, marked creatine kinase elevation, absence of cutaneous findings, aldolase of 1,002.3 U/L, and positive anti-SRP antibodies supported a suspected diagnosis of anti-SRP-positive IMNM. Further workup with muscle biopsy, electromyography, and magnetic resonance imaging was deferred during the critical illness and planned for outpatient evaluation. Although the likely diagnosis was clinically and serologically supported, further workup would be beneficial to confirm the diagnosis.

**Figure 1:**
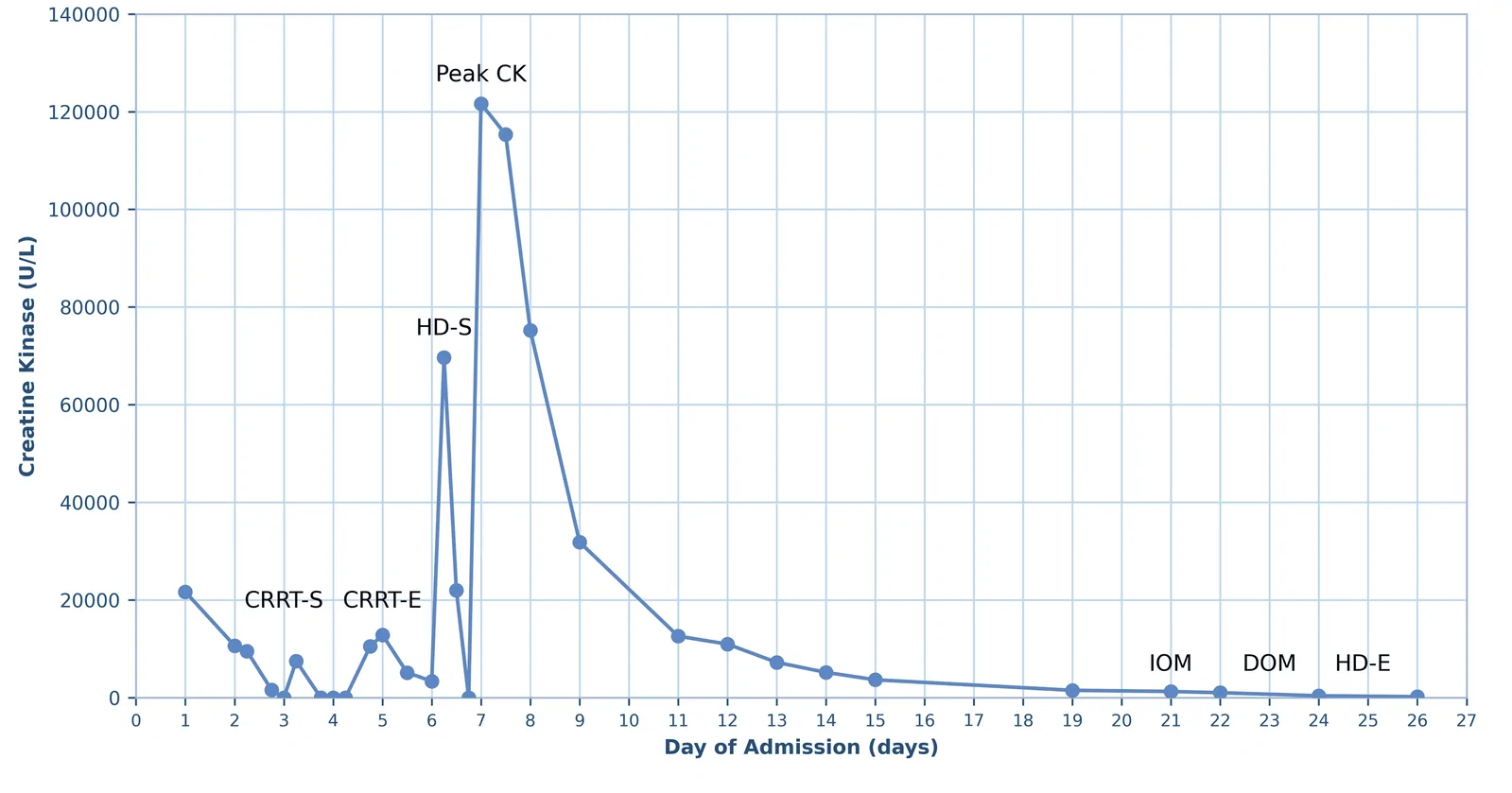
Creatine kinase trend during inpatient admission U/L: units per liter; CRRT-S: continuous renal replacement therapy started (day 3); CRRT-E: continuous renal replacement therapy ended (day 5); HD-S: hemodialysis started (day 6); HD-E: hemodialysis ended (day 23); peak CK: peak creatine kinase (day 6); IOM: initiation of methylprednisolone (day 21); DOM: discontinuation of methylprednisolone (day 23)

The patient received intravenous methylprednisolone 1000 mg IV daily for three consecutive days. Creatine kinase peaked at 121,627 U/L and subsequently declined to 227 U/L, accompanied by substantial improvement in strength (Figure 1). Intravenous immunoglobulin was considered but deemed unnecessary due to substantial clinical and biochemical improvement. The patient was weaned off hemodialysis upon improvement of creatine kinase and urine output.

The hospitalization was further complicated by acute pancreatitis with a peak lipase of 436 U/L (reference range: <160 U/L), an ultrasound-confirmed left upper-extremity deep venous thrombosis treated with heparin and apixaban, and severe anemia with a nadir hemoglobin of 6.2 g/dL (reference range: 13.5-17.5 g/dL) requiring blood transfusion. Intermittent fevers and leukocytosis with a peak white blood cell count of 35.2 × 10³/μ L (reference range: 4.0-11.0 × 10³/μ L) prompted bone marrow biopsy and flow cytometry, which showed no hematologic abnormality.

After administration of the high-dose corticosteroid regimen, the patient demonstrated marked improvement in strength and functional status. Occupational therapy was focused on fine motor coordination, balance, gait training, lower extremity strengthening, and endurance mobility. At the time of discharge, the patient scored a muscle rating of 4/5 in both upper and lower extremities, was able to ambulate without an assistive device, and could independently complete all activities of daily living. Further immunomodulatory therapy was deferred for outpatient follow-up due to improvement of symptoms. The patient was discharged to home health physical therapy and close follow-up with nephrology, rheumatology, and hematology.

## Discussion

Anti-SRP antibody-positive IMNM is a rare and often severe autoimmune myopathy characterized by rapidly progressive symmetric proximal weakness, marked creatine kinase elevation, and muscle fiber necrosis with minimal inflammatory infiltrate [6,8]. Diagnostic assessment typically integrates clinical findings, myositis antibody testing, electrodiagnostic studies, magnetic resonance imaging, and, when feasible, muscle biopsy [12-14]. Because corticosteroid monotherapy is frequently insufficient for sustained disease control, treatment often includes glucocorticoids combined with additional immunomodulatory therapy [15-17].

This patient’s presentation of the likely diagnosis was unusual for several reasons. First, severe rhabdomyolysis with oliguric acute kidney injury requiring continuous renal replacement therapy and hemodialysis was the dominant early manifestation. Renal complications in inflammatory myopathies may result from myoglobin-associated tubular injury, but the incidence, mechanisms, and long-term outcomes of renal disease in IMNM remain poorly defined [9]. The severity of this patient’s renal dysfunction and metabolic derangements also limited completion of a comprehensive inpatient myopathy evaluation.

Second, the patient became symptomatic at 23 years of age, which is decades younger than the typical age range of presentation. Younger age at onset has been associated with greater disease severity and an increased risk of refractory disease, which may be consistent with this patient’s rapid clinical decompensation, profound creatine kinase elevation, and severe renal complications [10,11].

Lastly, although corticosteroid monotherapy was sufficient to stabilize this patient during hospitalization, IMNM often requires maintenance immunosuppression, intravenous immunoglobulin, or B-cell-directed therapy to prevent relapse and improve long-term strength [15-17]. Close outpatient surveillance and additional testing were strongly recommended, particularly given the patient’s young age and severe initial presentation.

Alternative diagnoses were considered, including severe influenza-associated viral rhabdomyolysis, inherited or metabolic myopathies, inflammatory myopathies, vasculitis, and anti-glomerular basement membrane disease. However, isolated viral rhabdomyolysis was considered less likely given the extreme creatine kinase elevation, persistent symmetric weakness, and anti-SRP antibody positivity. Negative autoimmune, vasculitis, and other serologies made several overlapping inflammatory and renal autoimmune conditions less probable, but these diagnoses could not be ruled out due to the absence of histologic evidence.

It is likely that the acute influenza A infection may have acted as an unmasking event for the myopathy or a confounder for the initial rhabdomyolysis. Although viral rhabdomyolysis was considered, the extreme elevation in creatine kinase, persistent symmetric weakness, anti-SRP antibody positivity, and rapid response to high-dose intravenous methylprednisolone supported an underlying immune-mediated process. Additionally, the temporal association between the viral infection and symptom onset may have been coincidental rather than causally related to the underlying disease process.

Further work-up for myopathy was deferred during the patient’s critical illness and was not completed during the inpatient admission. Records of subsequent outpatient follow-up and completion of diagnostic studies are not readily available, and this gap remains a limitation of the report. Therefore, the case represents suggested IMNM supported by the clinical presentation and anti-SRP antibody positivity rather than histopathologic or electrodiagnostic confirmation.

This case reinforces the need to consider IMNM in young patients with severe nontraumatic rhabdomyolysis, particularly when weakness persists after treatment of an apparent infectious trigger. Earlier recognition may facilitate timely rheumatologic, nephrologic, rehabilitation, and neuromuscular evaluation before potentially irreversible complications develop.

## Conclusions

This case highlights an atypical presentation of probable anti-SRP-positive IMNM in a previously healthy young adult presenting with influenza A infection and fulminant rhabdomyolysis. Severe renal dysfunction and metabolic complications can obscure the underlying autoimmune process, especially when acute viral symptoms dominate the initial presentation. It is unclear to what extent the viral infection was related to these symptoms; however, there was an established temporal relationship. Regardless of the trigger, the persistence of symmetric muscle weakness and disproportionately elevated muscle enzymes should prompt consideration of an inflammatory or immune-mediated myopathy.

Early recognition of disease allows clinicians to initiate appropriate immunotherapy and coordinate multidisciplinary follow-up for renal recovery, rehabilitation, and long-term disease management. This case is limited by the lack of histopathologic, electrodiagnostic, and imaging confirmation during the acute hospitalization, as well as limited long-term follow-up. Further study is needed to identify early symptoms and potential complications, clarify triggers of disease, and develop optimal maintenance therapy for young patients with suspected anti-signal recognition particle-positive IMNM.
